# Ten simple rules for writing compelling recommendation letters

**DOI:** 10.1371/journal.pcbi.1008656

**Published:** 2021-02-25

**Authors:** Jennifer H. Kong, Latishya J. Steele, Crystal M. Botham

**Affiliations:** 1 Departments of Biochemistry and Medicine, Stanford University, Stanford, California, United States of America; 2 Office of Graduate Education, Stanford University, Stanford, California, United States of America; 3 Stanford Biosciences Grant Writing Academy, Stanford University, Stanford, California, United States of America; Carnegie Mellon University, UNITED STATES

## Introduction

Recommendation letters are necessary for advancement at all levels of academics, as they are widely required for grants and applications ranging from graduate school to tenured academic positions. As the name suggests, a recommendation letter is a written referral. However, writing an impactful recommendation that both stands out and voices genuine support for an applicant is a time-consuming task. The following tips will help you write more convincing recommendation letters that are NOT generic, resume-repeating, lacking substance, and doubt raisers [1]. To craft a compelling recommendation letter, discuss with the applicant the letter’s purpose and what they would like you to incorporate, ensure that you are the best person to write the recommendation, think deeply about the audience that will assess the letter, and take the time to craft a personalized letter that appropriately highlights the applicant’s fit for the award or position. The 10 simple rules outlined below work best if you know the applicant and their work well.

### Rule 1: Gather relevant information to assess if you can write a strong recommendation letter

If new to writing recommendation letters, start by collecting a few sample letters, which can be found on the internet. Ask your colleagues if they have any redacted examples that they are willing to share. Assess which recommendation letters stand out and use these as models.

#### Identify letter requirements

Before agreeing to write a letter, gather basic information from the applicant and ask: When is the letter due? What is the letter for? How is the letter submitted? Who will the letter be addressed to? What is known about the organization or individual that will receive the letter? Why was I chosen as a letter writer?

#### Respond with enthusiasm or decline

After gathering basic information about the needed letter, be honest and assess your ability to write a strong recommendation letter for the applicant. Do not hesitate to decline, if you can only provide a weak, generic, or unenthusiastic letter. In your assessment of your ability to write a strong recommendation letter, think about the following questions: Do I have enough time to write a strong letter? Am I the most appropriate reference? Have I observed specific traits/accomplishments that embody the needed qualifications for this opportunity? Can I sincerely, and with enthusiasm, recommend this applicant for this position or award?

#### Gather relevant information about the applicant

If you agree to write a recommendation letter, take time to learn more about the applicant. Ask the applicant for an updated biosketch or curriculum vitae (CV) to gather detailed information about their training and publications. Meet with the applicant (either in person, video conference, or by phone) to discuss their goals and career objectives. In addition, use this meeting to discuss the content of the letter. Ask what accomplishments or other information the applicant would like you to highlight. Develop a sense of the skills that you can emphasize to promote the applicant for the job, fellowship, or award they are applying for. The more information you gather about the applicant, the better equipped you will be to write a personalized letter that highlights their strengths and sincerely voices your support for their application.

Some letter writers ask the applicant to provide a draft letter as a starting point or recommend a joint effort in writing letters [2]. The practice of ghostwriting letters may seem like it saves time but can be problematic, especially when the letters lack your authentic language and voice [3,4]. Instead, ask for a bulleted list of points that the applicant thinks you should highlight in your letter.

### Rule 2: Allow sufficient time to write

Once you have agreed to write the letter, add the due date to your calendar and commit time to completing the task. Even for the most experienced letter writers, the creation of a strong and thoughtful letter for a new individual takes time. Expect the process to take 1 to 2 hours, potentially longer. To avoid last minute panic writing, create reminders for yourself. In addition, for the mental well-being of the applicant, submit the letter ahead of the deadline and notify the applicant when the task has been completed.

### Rule 3: Provide context for why you are a suitable letter writer

After a short introductory paragraph summarizing the applicant’s skills and qualifications for this opportunity, describe what makes you qualified to write the recommendation letter. This description of your prior experiences and relationship with the applicant will set the stage for the reader to trust your judgement. Remember, the letter is not about you so don’t go overboard, but you need to communicate to the reviewer that you are a credible source and possess the credentials necessary to accurately assess the applicant. The description of your qualifications should vary based on the context of the letter, for instance, listing your own awards may be appropriate when nominating a MacArthur Fellow but less so when writing a letter for an undergraduate applying to medical school. In describing your relationship with the applicant, include positive details regarding the length of time you have known the applicant, how extensively you have worked with the applicant, and any significant growth observed over the duration of your relationship.

### Rule 4: Address the requirements needed to be successful

In the main body of the letter, describe why you are recommending this applicant and elaborate on the applicant’s strengths for this opportunity. Focus on describing concrete accomplishments and examples of core competencies, for example, discipline-specific knowledge, communication skills, leadership, and management skills. Many professional societies focused on training have generated core competencies relevant for their trainees, for example, the National Postdoc Association core competencies [5] and Association of American Medical Colleges entry-level medical school competencies [6]. Refer to the applicant’s biosketch or CV to highlight accomplishments that set them apart from the applicant pool and make them the ideal candidate. For example, if the recommendation letter is for a research grant application, then focus on the applicant’s research accomplishments. In the letter’s middle paragraphs, describe accomplishments/technical competencies, like how the applicant developed an assay, used this assay to make novel discoveries, and published these findings in peer-reviewed journals. Conversely, if the recommendation letter is for a managerial position, then focus on the applicant’s abilities to lead a research team. Again, focus on accomplishments relevant for this opportunity, for instance, the applicant led productive team meetings, played a critical role in synthesizing data from multiple researchers, and mentored a summer student on a project that was ultimately published. Whether the recommendation letter is for a grant or job application, remember to tailor the letter to address the needs of the application.

### Rule 5: Be memorable by adding illustrative anecdotes

In your evaluation of the applicant, abstain from using generic praises or cliché statements like “accomplished student” or “highly motivated learner” without also describing a specific example illustrating that characteristic. Outline memorable stories that illustrate the applicant’s successes, as suggested by Dino Di Carlo, Professor of Bioengineering at University of California, Los Angeles [7]. As with any good story, create a narrative that includes background or context, introduces a turning point or conflict, and concludes with a resolution.

Use strong descriptors associated with traits you want to highlight (Table 1) but mindful of your word choices. Just as we write and then edit to fix grammatical errors, it is equally important to write and then edit to identify and minimize bias. By definition, implicit bias or unconscious bias is difficult to detect because the perceptions and stereotypes from which they arise can be outside our own conscious awareness. In academic recommendation letters, bias often surfaces inadvertently through the use of stereotypical descriptors. While we recognize that there are many other types of biases (e.g., affinity/similarity bias, conformity bias, halo or horns (seeing people as angels—positively or negatively), ageism, beauty), we focus here on recommendations to address gender and racial biases as these are common types of bias in recommendation letters. Moreover, the strategies presented here can be informative in addressing other biases. These descriptors are by no means a comprehensive list but seek to provide some awareness behind word choice and its consequences.

**Table 1:** Shortlist of superlatives to elevate descriptions of accomplishments or competencies.

Analytical /Critical Thinking	Innovative /Creative	Productive	Leadership	Enthusiasm /Vigor
Robust	Unprecedented	Accomplished	Independent	Eager
Methodical	Ingenious	Proactive	Adaptable	Optimistic
Investigated	Inventive	Systematic	Trustworthy	Zealous
Calculated	Pioneered	Results oriented	Spearheaded	Self-driven
Logical	Trailblazer	Resourceful	Enterprising	Energetic

Groups across multiple disciplines have studied recommendation letters by closely analyzing the words used to describe the candidate [8–13]. Remarkably, all of these studies concluded that the words used to describe women and minorities are often quantitatively different than the words used to describe men. Women and minorities are more frequently described as communal or compassionate using words like “unselfish,” “warm,” “helpful,” “kind,” “interpersonal,” “sensitive,” or “caring.” These words are not bad or negative, in fact they are highly valued traits in society, but these words can be associated with negative hiring decisions and if used, should be done so carefully to avoid unconscious bias. Women and minorities are also frequently described using grindstone adjectives like “tireless,” “committed,” or “hardworking,” which evoke effort but not concrete skills. Lastly, letters written for women and minorities frequently lack repeated superlatives like “superb,” “outstanding,” “exceptional,” “excellent,” or “rising star”. We recommend using a bias calculator for feedback on your word choice [14] and completing bias training to become more aware of how bias can influence your writing. Print out and keep these tip sheets [15–17] on hand as well to avoid gender and/or racial bias. Lastly, be mindful that stereotypes are not limited to gender and race. Bias shades our perception of age, disabilities, and socioeconomic status. To learn more about the many biases that exist and how to avoid perpetuating these beliefs through your writing, we highly recommend reading the Bias-Free Language Guidelines published by the American Psychological Association (APA) [18].

### Rule 6: Quantify by comparing applicant to peers

We are taught to value individuality and appreciate that each researcher comes with a unique set of skills and insights. As a result, ranking people feels strange and uncomfortable. However, comparing an applicant to their peers is an important way to quantify and communicate their fit for the opportunity at hand. This comparison can be done casually by describing the applicant as “one of the best graduate students I have trained in their ability to integrate ideas from diverse fields into new research avenues.” Or the applicant can be elevated even further by providing just a little more detail. For example, describing the applicant as “among the top 1% of students I have trained in the past 10 years.”

### Rule 7: Keep it professional

Avoid including details about the applicant’s personal life without verifying it with the applicant. It may seem helpful to provide context about obstacles the applicant has overcome, but be conscious that the applicant may not want that information disclosed. Also, be aware that mentioning the applicant’s affiliations with certain groups may reveal information about their identity that they don’t want discussed. Generally speaking, avoid topics such as the following without first discussing it with the candidate: mental illness, gender, race, nationality, religion, age, appearance, physical or mental handicaps, marital status, parental status, or any political point of views. Avoid descriptions of individuals, primarily based on personality characteristics, that reinforce stereotypes about gender, race, and/or other identity characteristics (refer to Rule 5). If you want to include potentially sensitive information, ask the applicant for permission, but be aware that reference letters for females are more likely to mention irrelevant personal life information [19].

During your information gathering step (see Rule 1), the applicant may reveal information that they specifically would like conveyed in the letter. For example, as the letter writer, it can be helpful if you explain an unproductive research period in the applicant’s PhD because of the need to switch mentors as a fourth-year graduate student. Or that the applicant had extensive caregiving responsibilities for a younger sibling as an undergraduate which affected their grades during sophomore year. Mentioning these types of situations in your letter can communicate these circumstances without making the applicant sound like they are making excuses.

Keep the tone of the letter positive and avoid inserting unnecessary doubt in an effort to make the letter sound more sincere, such as using “although” or “even though.” For example, rephrase “Although the applicant came in with very little research experience in Field A, their background in Field B has transformed our research” to “This applicant’s expertise in Field B has transformed our research focus in Field A in the following ways …” Don’t use ambiguous language, like “I assume…”, or any statements that lack specificity. Honesty is essential in a recommendation letter, so offset any potentially perceived weaknesses with positivity and/or stories of overcoming academic or research challenges.

### Rule 8: Maintain a level of high enthusiasm

Generally speaking, longer letters are perceived as more enthusiastic [13]. Systematically studied letters of recommendations have revealed that female applicants’ letters are generally shorter in length [19]. Don’t go overboard but aim to get each recommendation letter on to the second page, unless otherwise limited by the instructions.

In writing a recommendation letter, you have committed to helping your colleague or trainee. Presumably, you will spend time crafting this letter because you want the applicant to be successful and you believe the applicant deserves what they are applying for. So, remember to express this enthusiasm in your letter when describing both the applicant’s work and their future prospects. This is not the time for modesty, if appropriate include statements like “the applicant has conducted groundbreaking research in the lab and I am excited about their future studies” or “the applicant is an exceptional young scientist with a stellar academic future as an independent investigator and scientific leader.” Additional useful phrases to use in recommendations are listed here [20].

### Rule 9: Express your willingness to help further

In the concluding paragraph of the reference letter, express an eagerness to help further if there are additional questions. Make sure to provide your contact information in the form of an email address and/or phone number. As stated in Rule 7, avoid raising any unnecessary doubts about the applicant. However, if doubts are raised in the letter, then it is equally as important to provide an avenue for clarification and an opportunity for follow-up.

### Rule 10: Edit and proofread

When writing any document, we tend to get too close. If time allows, put the letter aside, create some distance between you and your writing, then come back to it with a fresh set of eyes. This technique will help you improve the letter. In addition, during this break your brain will unconsciously think about the letter, latent memories or experiences will surface, and these additional insights can be added to further personalize the letter and make it stronger.

With the text of the letter finalized, make the letter look professional by using your institution’s letterhead, as appropriate, and adding your signature. If possible, include a personalized salutation instead of something general like “to whom it may concern.”

### Note to applicants: Instructions for getting great recommendation letters

Having been on both the giving and receiving side of the recommendation letter process, we can confidently say that the applicant has the power to significantly contribute to the strength of their own letter [21]. As an applicant, request a letter at least a month in advance to provide your referee with time to craft a strong letter. Set up a time to meet with your referee and discuss the contents of the letter, specifically why you chose your referee and what you would like them to address in the reference letter. Provide your referee with all the information necessary to write a strong letter; this includes an updated biosketch or CV, detailed letter instructions, and a personal statement that addresses your academic and professional goals. If the letter needs to be mailed, provide an envelope and postage. Lastly, send a friendly reminder to your referees as the due date approaches. Afterwards, send a thank you note to your referees and let them know how it turned out.
